# Design of Muscle Reflex Control for Upright Standing Push-Recovery Based on a Series Elastic Robot Ankle Joint

**DOI:** 10.3389/fnbot.2020.00020

**Published:** 2020-04-28

**Authors:** Yuyang Cao, Kui Xiang, Biwei Tang, Zhaojie Ju, Muye Pang

**Affiliations:** ^1^School of Automation, Intelligent System Research Institute, Wuhan University of Technology, Wuhan, China; ^2^Intelligent System & Biomedical Robotics Group, School of Computing, University of Portsmouth, Portsmouth, United Kingdom

**Keywords:** muscle reflex, ankle joint, upright standing, push-recovery, series elastic actuator

## Abstract

In physical human–robot interaction environment, ankle joint muscle reflex control remains significant and promising in human bipedal stance. The reflex control mechanism contains rich information of human joint dynamic behavior, which is valuable in the application of real-time decoding motion intention. Thus, investigating the human muscle reflex mechanism is not only meaningful in human physiology study but also useful for the robotic system design in the field of human–robot physical interaction. In this paper, a specialized ankle joint muscle reflex control algorithm for human upright standing push-recovery is proposed. The proposed control algorithm is composed of a proportional-derivative (PD)-like controller and a positive force controller, which are employed to mimic the human muscle stretch reflex and muscle tendon force reflex, respectively. Reflex gains are regulated by muscle activation levels of contralateral ankle muscles. The proposed method was implemented on a self-designed series elastic robot ankle joint (SERAJ), where the series elastic actuator (SEA) has the potential to mimic human muscle–tendon unit (MTU). During the push-recovery experimental study, the surface electromyography (sEMG), ankle torque, body sway angle, and velocity of each subject were recorded in the case where the SERAJ was unilaterally kneed on each subject. The experimental results indicate that the proposed muscle reflex control method can easily realize upright standing push-recovery behavior, which is analogous to the original human behavior.

## Introduction

The mechanical ability of the ankle joint to stand upright steadily is of great significance in human daily lives. However, due to the high center of mass (about 64% of body height aboveground) and the disproportionately small supporting feet, it is not easy for human beings to stably maintain upright stance (Roberts, 2002). So far, it has been discovered that the reflex control mechanism plays an important role in guaranteeing the outstanding performance of ankle joints in standing in the presence of external disturbances (Loram and Lakie, 2002). The reflex component contributes 10–40% resistance torque to the gravity destabilizing effect (Vlutters et al., 2015), and it may reflect the potential mechanism of upright stance postural sway. Maintaining upright stance is a fundamental and challenging task for wearable robotic devices, especially for powered ankle joint prosthetics (Buckley et al., 2002; Emmens et al., 2018). Studying the muscle reflex control on human upright standing push-recovery not only promises to increase visibility of ankle joint dynamic properties for decoding motion intention but also is meaningful to achieve a bio-inspiration-based control method for the wearable robotic system in the field of human–robot interaction control.

Human upright standing push-recovery results from additive interactions of the senses, including vestibular, tactile, and proprioception, under the neuromuscular control mechanism. The muscle reflex, such as stretch reflex, is independent from cortical involvement and works as the most basic control mechanism of the central nervous system (CNS). Hence, the muscle reflex has relatively short afferent and efferent transmission delays, which consequently enhances the fast response ability of human muscle to external disturbances. The muscle reflex alone is powerful to maintain human upright balance during quiet standing in the case where only small environmental interference is considered (McMahon, 1984). In the muscle reflex control mechanism, body sway velocity and angle have profound effects on ankle extensor activities during quiet stance (Masani et al., 2003). During human maintaining upright balance, body sway kinematics contains motion information on the sequence state of body for real-time decoding motion intention. Apart from the negative feedback, such as the negative angle feedback and the negative velocity feedback, some researchers suggested that the positive feedback also appears in the muscle reflex and can provide stable load compensation during human locomotion (Prochazka et al., 1997). Despite the muscle reflex alone is able to compensate small disturbances, a feed-forward mechanism modified by the cortical involvement can considerably improve human balance recovery ability even under strong disturbances since it can change the muscle reflex gains during push-recovery movement (Fitzpatrick et al., 1992). Consequently, the behavior of ankle can be varied from stiff to compliant so as to make it alterable under different environmental interactions.

Since the muscle reflex mechanism can not only provide insights to the study on human ankle joint properties but also suggest clues to the intelligent control of bionic robots physically interacted with humans to maintain upright stance, it has aroused extensive research interests of different researchers from the fields of human physiology and robotics. Winter et al. (1998) have proposed a stiffness model for quiet standing. In their work, muscles are assumed to act as springs and can create restoration torque determined by the ankle joint stiffness when body sways deviate from the desired position. As an extension, simple ankle joint stiffness measurement results have been provided to support the “stiffness control” assumption (Winter et al., 2001). In order to maintain balance during human bipedal quiet stance, Masani et al. (2006) have presented a feedback proportional-derivative (PD) controller to efficiently generate a desired preceding motor command. Later on, Loram et al. (2007) have proven that a constant stiffness could be insufficient to maintain upright stance balance. For the sake of meeting higher control requirement, Vallery et al. (2008) have designed the compliant actuation and assistance as needed (AAN) algorithms applied in rehabilitation robots for neural recovery of locomotion, where the apparent mechanical impedance of devices is programmable to achieve the desired interaction control. In 2013, Rouse et al. (2013) have elucidated the stiffness and quasi-stiffness and also clearly interpreted the differences and similarities between these two concepts in the context of biomechanical modeling. The muscle reflex control is one of the paramount elements composing the mechanism of apparent angle–torque regulation relationship during dynamic movement.

Developing a bio-inspiration-based controller to maintain upright stance in the present of external disturbance for a unilateral prosthetics is meaningful as the robotic device should not only provide enough recovery torque but also follow the regulation mechanism of human to achieve a successful cooperation movement (i.e., to pull the torso back to the balance state together with the contralateral lower limb). Aiming at effectively achieving human upright standing push-recovery, this paper first proposes a specialized ankle joint muscle reflex control algorithm. Afterward, the proposed control algorithm is employed on a self-designed compliant actuation device which is named series elastic robot ankle joint (SERAJ) to mimic the muscle–tendon unit (MTU). Lastly, the feasibility of the proposed control algorithm is verified *via* experiments from four aspects: relationship between ankle torque and sway angle, stability faced with external disturbances, influence on the other ankle muscles, as well as ankle joint dynamic properties during upright standing push-recovery. The experimental results reveal that the proposed reflex control algorithm can easily achieve human upright standing push-recovery, analogous to the original human behavior.

The remainder of this paper is organized as follows. The *Muscle-Tendon Unit and Muscle Reflex Control* section introduces MTU and human muscle reflex for push-recovery. The mechanical design of the SERAJ is stated in the *Mechanical Design of Robot Ankle Joint* section. The *Reflex Control Strategy* section describes the proposed reflex control strategy. The experimental tests and results are shown and analyzed in the *Experimental Setup* and *Experimental Results* sections, respectively. The last section concludes this study and shows some future works pertaining to this paper.

## Muscle–Tendon Unit and Muscle Reflex Control

The function of MTU during locomotion and the schematic of muscle control for upright standing push-recovery are stated in the hereafter contents of this section due to the fact that they contain important inspirational functions for the mechanic design of the compliant robot ankle joint and reflex control strategy.

### Compliance of Muscle–Tendon Unit

In addition to the muscle function, the tightly integrated and complementary function of tendon remains necessary and essential for generating joint torque and power during human push-recovery movement. The mechanical role of MTU provides a key energy-conserving mechanism that the elastic energy storage and recovery process is achieved in tendon from the body movement (Cavagna et al., 1964; Alexander and Bennet-Clark, 1977; Alexander, 1984). MTU exhibits significant viscosity due to the tendon's elastic behavior, where the length of tendon varies in proportion to the applied load. This implies that the muscle and tendon can be regarded as the torque generation element and compliance element, respectively. This combination is the original design idea of series elastic actuator (SEA).

The ability to enhance muscle performance of MTUs depends on the mechanical behavior of compliant structure, where energy store is an important ability and forms the fundamental mechanism of movement dynamic behavior (Alexander and Bennet-Clark, 1977). In a so-called “fixed-end contraction” (Jacobs and Horak, 2007), the tendon compliance is expressed in terms of its potential influence on muscle fiber shortening. In such a case, MTU has a tendency to expend a large fraction of its shortening capacity on stretching tendon compared to muscle contractile elements (Jacobs and Horak, 2007). It has been demonstrated that MTU as an integrated actuator can exhibit promising performance for a wide range of locomotion activities (Jacobs and Horak, 2007). This indicates that the compliant MTU can be considered as a force-producing spring and its elastic mechanism acts as muscle power amplifiers by directly storing the work done by tendon stretching during human locomotion. This dynamic function of MTU is consistent with Winter et al.'s (1998, 2001) spring control model at the ankles.

### Muscle Reflex for Upright Standing Push-Recovery

Many elaborate impedance control-based algorithms have been proposed for powered prosthesis to achieve movement ability such as locomotion, up-down stairs, and sit to stand. However, upright standing push-recovery during small balance (i.e., using ankle strategy) is quite different. There is much less movement involved in ankle strategy standing balance. Although inconspicuously, muscle reflex compensation is necessary as intrinsic stiffness is not enough to keep upright stance balance (Loram et al., 2007). Furthermore, it is useful to utilize the inherent reflex control mechanism of human to fulfill a human-like behavior to realize a reliable push-recovery movement, cooperating with the contralateral lower limb.

**Figure 1:**
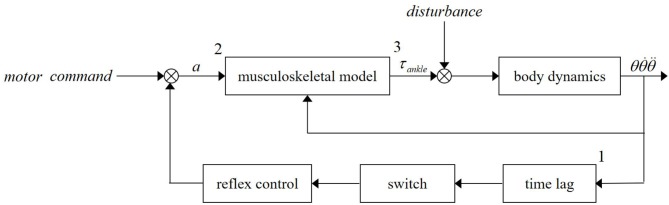
Upright standing push-recovery model with reflex control.

Based on the research work done by Fitzpatrick et al. (1996), the schematic of upright standing feet-in-place push-recovery is presented in Figure 1. In this figure, “*a*” is muscle activation level. “τ_*ankle*_” denotes ankle torque. “*Disturbance*” stands for the external force exerted on backs of subjects. The “*musculoskeletal model*” includes Hill-type muscular model and muscle-skeleton anatomy structure. Information of muscle torque arm can be obtained from the anatomy structure. “*Reflex control*” generally includes proprioceptive, visual, and vestibular reflexes. Only muscle reflex is considered in this study. “*Time lag*” contains reflex pathway and muscle biomechanical dynamic time lag. The motor command stems from high-level nervous system where the cerebral cortex is involved. When external disturbances occur, the reflex control loop (maybe together with some motor commands) activates muscles around the ankle joint, so that torques pulling the body back to an upright posture can be yielded. Note that the block “*switch*” in Figure 1 is designed to trigger different muscle reflexes.

It is worth mentioning that the push-recovery schematic displayed in Figure 1 can be separated into two parts, that is, the muscle activation generation part (from 1 to 2) and the ankle torque generation part (from 2 to 3). The first part describes the generation mechanism of muscle activation by muscle reflex. The second part depicts the impacts of the muscle activation and ankle joint on the ankle torque. Muscle activation level is the connection of these two parts and can be calculated by surface electromyography (sEMG).

During human upright standing push-recovery movement, muscle reflex responds to joint or body kinematics/kinetics state variations. Muscle spindle is sensitive to the fiber length change and its change rate. When muscle is stretched, the spindle generates corresponding bio-electrical signal transmitting to spinal cord. Spinal cord generates feedback signals to activate muscle fibers. Muscle fibers contract to against the stretch. The following PD-like mathematic form is often used to describe this function (Fitzpatrick et al., 1996):

(1) $$\begin{array}{l} a\left(t\right)=a_{0}+p_{a}\left(L_{CE0}-L_{CE}\right)+d_{a}V_{CE} \end{array}$$

where *a*_0_ is the initial muscle activation level. *L*_*CE*0_ and *L*_*CE*_ represent fiber optimal and current lengths, which are, respectively, calculated from the ankle torque generation part and the ankle joint angle. *V*_*CE*_ indicates the fiber length changing rate and is replaced by ankle joint angular velocity for simplicity in the following reflex controller design.

Many factors affect muscle reflex control, such as visual and vestibular processing system (McMahon, 1984). However, these factors influence more likely the reflex gains, rather than the form (Welch and Ting, 2008). In this study, we try to keep influences from visual and vestibular system unchanged by using a constant eye-open operation condition and applying a “ball release” disturbance in which acceleration of the head is small. In this case, the main factor involving reflex control is the ankle joint state. This disturbance is very common in daily lives, such as pushing a light door or holding a small stuff in the arm. For more details about contributions of different reflexes on upright standing push-recovery, the reader can refer to Peterka (2002) and Jacobs and Horak (2007).

This paper selects and applies the sEMG signals of SOL and GAS to quantify the muscle activation level because these two muscles have been found to play important roles in human push-recovery movement. When converting sEMG signals into the muscle activation level, this paper adopts a commonly used method proposed by Jacobs and Horak (2007) to process the raw sEMG signals of the three targeted muscles. The process method can be summarized as follows: (1) a fourth-order high-pass Butterworth filter with cutoff frequency of 10 Hz is then used to remove direct current noise; (2) the reprocessed signals are rectified; (3) a low-pass Butterworth filter with cutoff frequency of 5 Hz is finally applied to obtain the envelope *e*(*t*).

After processing the raw sEMG signals following the method stated above, the muscle activation level *a*(*t*) is calculated as follows:

(2) $$\begin{array}{l} u\left(t\right)=\alpha e\left(t-d\right)-\beta_{1}u\left(t-1\right)-\beta_{2}u\left(t-2\right) \end{array}$$

(3) $$\begin{array}{l} a\left(t\right)=\left(e^{Au\left(t\right)}-1\right)/\left(e^{A}-1\right) \end{array}$$

where Equation (2) represents the muscle activation dynamics, and Equation (3) is used to non-linearize the results. Equation (2) represents a discretized second-order relationship between e(t) and u(t), which is the dynamic between muscle activation and muscle force generation. According to Buchanan et al. (2004), it needs to guarantee that Equation (2) is critically damped and stable. In this paper, parameters α, β_1_, and β_2_ are set as 2.25, 0.5, and 0.5 following our previous study (Pang et al., 2019). *d* is the electromechanical delay and set to be 10 ms as reported in Corcos et al. (1992). Equation (3) is used to non-linearize muscle activation results. Parameter *A* could be set to be a constant in the range of [−3,0]. Moreover, according to our pilot test, the amplitude of muscle activation would exceed 1, which is not allowed as the range of muscle activation is from 0 to 1 in the case where *A* is set too small. To obtain a little non-linear effect, *A* is empirically set to be −0.5 in this paper. It is notable that different settings of these parameters can affect the relationship between muscle activation and muscle force. However, this effect can be regulated by a constant or variable gain in robotic control frame, which is to say the trend extracted by Equations (2, 3) is more meaningful than the amplitude in this work.

Hill-type muscular model is implemented to calculate tension of MTUs. Tensions of MTUs are summed to predict ankle torque. This paper borrows the conventional form described in Fitzpatrick et al. (1996) to calculate the tensions of MTUs. There are two parts in the conventional form which are, respectively, the passive serial element and the contractile element parts (Buchanan et al., 2004, 2005). The passive serial element part (SE) represents the muscle-tendon elastic property. The contractile element part is composed of a contractile element (CE) and a passive element (PE), representing the muscle fiber active and the passive force properties, respectively. The calculation of muscle-tendon *F*_*MTU*_ force can be mathematically given by:

(4) $$\begin{array}{l} F_{MTU}=F_{T}=\left(F_{CE}+F_{PE}\right)\text{cos }\theta_{p} \end{array}$$

where *F*_*T*_ is the tendon tension. *F*_*PE*_ and *F*_*CE*_ are the passive and the active forces generated by muscle fiber, respectively. θ_*p*_ indicates the current pennation angle. Note that θ_*p*_ can be regarded as constant in this study since the variation of ankle joint angle is small enough.

Moment arms (*r*) of different muscles are obtained from OpenSim model^1^ and linearly scaled by the height of each subject. As ankle joint angle changes in a small range (within 0.1 rad), values of moment arms are set as constants. Then, the total ankle joint torque can be computed as follows:

(5) $$\begin{array}{l} F_{T}=\left[F_{\text{max}}/e^{S}-1\right]\left[e^{\left(SL/L_{\text{max}}\right)}-1\right] \end{array}$$

where *F*_*max*_ is the maximum muscle fiber force exerted at optimal fiber length. *S* is a shape parameter. *L* and *L*_*max*_ are the current length and the slack length of the tendon, respectively.

Covariance matrix adaptation-evolution strategy (CMA-ES) optimization method (Hansen, 2016) was used to find the proper parameters of the model. Under the ankle-strategy condition, the body can be regarded as a first-order inverted pendulum and the dynamic equation can be expressed as:

(6) $$\begin{array}{l} I\ddot{\theta }=\tau_{ankle}-mgI\text{ sin }\theta -\tau_{e} \end{array}$$

where τ_*ankle*_ is ankle torque. τ_*e*_ denotes disturbance torque. *I* and *m* are the inertia and mass of the body, respectively. Ankle joint angle θ can be read from the angular transducer or the motion capture system.

## Mechanical Design of Robot Ankle Joint

**Figure 2:**
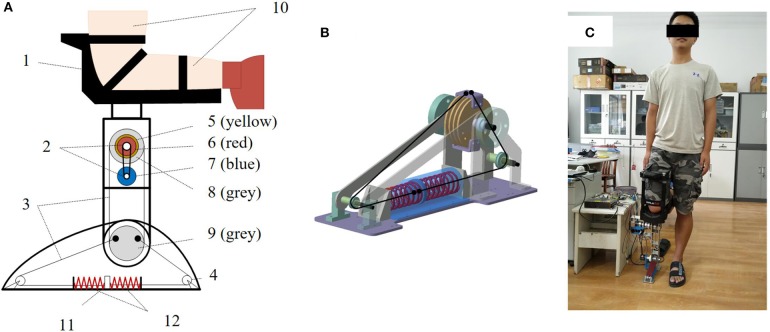
Design of the series elastic robot ankle joint (SERAJ). **(A)** Structural schematic of SERAJ: (1) iWalk2.0 Hands Free Crutch, (2) belt pulley, (3) steel cables, (4) pulley, (5) coupling, (6) reducer, (7) motor, (8) shank-rotary-disk (SRD), (9) foot-rotary-disk (FRD), (10) experimenter's lower limb. **(B)** Design of the series elastic actuator (SEA): Steel cables connect the springs to the actuator, which is detached from the ankle joint. **(C)** Photographic impression of SERAJ in operation.

In order to evaluate the proposed reflex control algorithm, there exists a necessity to design a wearable robotic device to mimic the human ankle joint neuromechanical properties. To this end, this paper designs a SERAJ. For guaranteeing the wearable suitability of the designed robotic device, the SERAJ device is designed with similar size to human lower limb (mechanical specifications are listed in Table 1). As visualized in Figure 2, the mechanical structure of the designed SERAJ mainly includes the foot, shank, and the ankle joint. The mechanical foot is designed as a support polygon for upright standing with similar size of human foot. The top of the mechanical shank is connected to a knee crutch [iWalk2.0 Hands Free Crutch (1 in Figure 2A)], which allows the device to be wearable on the knee of human. The mechanical ankle joint acts as a connection between the mechanical foot and shank. In order to minimize the inertia of the movement segments with a promising structural strength, all these three aforementioned parts are constructed from lightweight aluminum. The motor (7 in Figure 2A) and reducer (6 in Figure 2A), coupled by a belt pulley (2 in Figure 2A) as an integrated actuator, are positioned on the lateral mechanical shank surface, where the output end of reducer is connected to the shank-rotary-disk (SRD) (8 in Figure 2A) through a coupling (5 in Figure 2A). The rotation of the SRD drives foot-rotary-disk (FRD) (9 in Figure 2A) synchronously by steel cables (3 in Figure 2A), which further results in movement of slider (5 in Figure 2A), connecting with FRD *via* cables, to compress spring (6 in Figure 2A). Consequently, robot ankle joint is driven under the torque action, which allows bodies of wearers to sway back or forth in a very narrow range. In summary, the self-designed SERAJ is characterized by (1) a self-contained single degree-of-freedom (DOF) joint which allows for a 90° of rotation in the sagittal plane and (2) the intrinsic compliance to allow adaptable performance in external physical interaction.

**Table 1:** Mechanical specifications of the series elastic robot ankle joint (SERAJ) (where *F, S*, and *J* are the SERAJ's foot part, shank part, and joint part, respectively.

**Dimensions**	**F**	**l**	**23.8 cm**
		w	7.8 cm
	S	l	33.3 cm
		w	3.9 cm
	J	r	5 cm
	W	h	40 cm
Mass	W	3.3 kg	
Material	F	Aluminum allory	
	S	Aluminum allory	
	J	Stainless steel	
Range of Motion	F	−90° to 90°	
	S	Immovable	
	J	360°	
Degree of Freedom		Single	

*W, whole robot. l, w, r, and h, length, width, radius, and height, respectively*.

It is worth noting that the purpose of designing SERAJ is neuromechanical to provide a platform to test control algorithm which mimics human push-recovery strategy.

### Series Elastic Structure

In contrast to traditional stiff robotic joint design, whose actuator merely includes a motor and a reducer, the designed SERAJ is intrinsically compliant, similar to AMP-Foot 2.0 presented in Cherelle et al. (2012). The designed robotic device features the SEA proposed by Pratt and Williamson (Pratt and Williamson, 1995) to provide back-drivable ability and position-based force control with highly geared motor. As shown in Figure 2B, two compression springs are mounted as elastic elements and added between the reducer and the load to form the series elastic structure. The stiffness of the spring is 76.59 N/mm and the maximum compressed length is 16 mm. According to the mechanical design of SERAJ, the spring compression *l*_*s*_ can be calculated as follows:

(7) $$\begin{array}{l} l_{s}=\left(\theta_{a}-\theta_{r}\right)R \end{array}$$

where θ_*a*_ and θ_*r*_ are the rotation angle of ankle joint and rotation angle of reducer output shaft, respectively. *R* is designed to be 0.05 m in the SERAJ, denoting the radius of the FRD.

### Separate Structure

In terms of muscle reflex control, the actuators are required to be high-precision force sources (Vallery et al., 2008). To this end, mass and inertia of the actuated construction need to be minimized. However, the means of effectively reducing negative impacts of these problems *via* control technologies are quite limited since control technologies mainly concentrate on control stabilities rather than the mechanical structure of the device (Vallery et al., 2008). To handle this issue, we applied steel cables to achieve a more flexible transmission in the actuator in self-designed SERAJ, instead of the general rigid connecting rods. Thus, the motor and reducer in the actuator can be placed far from the ankle joint, which can reduce the physical dimensions of the ankle joint and weaken the negative effects of their mass and inertia, to a large extent. One of the two springs is under compression and tries to expand, so that the cables always stay in tension during operation. Table 2 reports the specifications of the actuator in our self-designed SERAJ.

**Table 2:** The specifications of the actuator in the designed series elastic robot ankle joint (SERAJ).

**Motor**	**maxon RE 40−148867**	
Torque Constant (motor)	30.2 mNm/A	
Spring Stiffness	76.59 N/mm	
Gear Ratio (reducer + belt pulley)	72: 1	
Nominal Torque	15.744 Nm	
Nominal Speed	105.287 rpm	
Stall Torque	174.24 Nm	
Power	Voltage	24 V
	Current (max)	5 A
Communication	RS-232	
Sensing	angular position	
	angular velocity	
	voltage	
	current	

## Reflex Control Strategy

**Figure 3:**
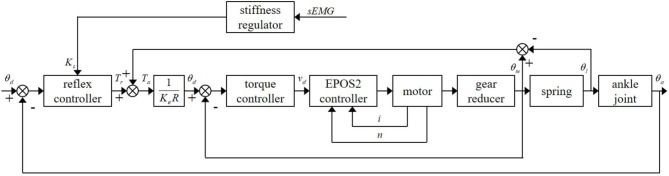
The control scheme of the proposed ankle joint muscle reflex control.

The proposed ankle joint muscle reflex control strategy is multilayered and includes five feedback loops, as well as one feed-forward loop. Multi-feedback closed loops consist of a reflex control loop, a torque control loop (position control loop), a speed control loop, and a current control loop. A feed-forward mechanism is used to change reflex gain in reflex control for adaptable compliance of SERAJ. The control scheme of the proposed ankle joint muscle reflex control strategy is shown in Figure 3. The control strategy of each loop in the proposed reflex control strategy is individually interpreted in the following contents of this section.

### Muscle Reflex Control Loop

In this paper, similar to the study conducted by Winter et al. (1998, 2001), human ankle joint is simplified as a single DOF joint in the sagittal plane and regarded as a pivot point. Once a human is subjected to an external disturbance in quiet standing, the center of mass (COM) deviates away from the equilibrium position, which results in an undesired torque on the body and causes the body to lean forward or backward. The undesired torque caused by COM shifting increases with the body sway angle increasing. To regain standing balance, muscles around the ankle joints contract to yield the restoration torque, so that impacts of body sway can be resisted during quiet standing. In this paper, the ankle joint angle is supposed to be equivalent to the body sway angle according to Gatev et al. (1999).

To mimic stretch reflex control, a PD-like form is adopted, in which the proportional part stands for the response of muscle spindle to muscle fiber length change and the derivative part represents the response to fiber length changing rate. According to the structure design of SERAJ, the deformation of spring, which can be calculated by the joint angle, is regarded as the muscle fiber length change. As a consequence, joint angular velocity represents the fiber length changing rate. A variation in ankle angular velocity indicates the direction and intensity of restoration torque at the ankle joint in the next time instant. The desired restoration muscle activation level used in the developed reflex control loop is given as follows:

(8) $$\begin{array}{l} A_{r}=K_{s}\left(\theta_{a}-\theta_{0}\right)+D{\dot{\theta }}_{a} \end{array}$$

where *A*_*r*_ is the restoration muscle activation level. *K*_*s*_ and *D* stand for the proportion and derivative reflex gains, respectively.

In order to provide a stable force compensation in the developed muscle reflex control framework, similar to Prochazka et al. (1997), a positive force feedback control loop is involved in this framework to compensate for the ankle torque as follows:

(9) $$\begin{array}{l} T_{a}=T_{r}+T_{c} \end{array}$$

where *T*_*a*_indicates ankle torque. *T*_*r*_ stands for the restoration torque produced by Equations (4, 5) using *A*_*r*_ as the input. *T*_*c*_ denotes compensation torque and can be obtained as:

(10) $$\begin{array}{l} T_{c}=CT_{s} \end{array}$$

where *C* represents the compensation coefficient, and *T*_*s*_is the mechanical torque generated by compression spring.

### Torque Control Loop

As stated above, a compression spring is added between the reducer output end and the load to increase the compliance of the self-designed SERAJ. Apart from this advance, such a structure design also increases the shock tolerance of the device and turns the torque control problem into a position control issue, so that the torque accuracy can be improved. Since the ankle torque in SERAJ is proportional to the spring compression multiplied by its spring constant and moment arm, the ankle torque can be obtained by:

(11) $$\begin{array}{l} T_{s}=K_{e}l_{s}R \end{array}$$

where *K*_*e*_ is spring constant and set to be 76.59 N/mm. *l*_*s*_ is the spring compression and can be gained *via* Equation (7).

During the application of the self-designed SERAJ, a maxon RE 40 motor driver is applied as the driving source. Since the maxon EPOS2 controller offers a fast and reliable way to automatically tune the regulation gains of the current and velocity and can easily adaptively adjust its parameters online, which is in full compliance with the control requirements of the maxon RE 40 motor, this controller is selected to control the speed and current of the motor applied in SERAJ.

### Stiffness Regulating Loop

During upright standing push-recovery, the ankle joint stiffness needs to constantly change with the variation of the compliant actuation. In this paper, we assume that each subject has the same stiffness on two ankle joints. Due to the SERAJ being equipped on the right knee of each subject, rather than the function of the right ankle in quiet standing, the stiffness of the left ankle joint is referred by SERAJ to adjust the stiffness or proportional gain in the reflex control loop. Recall that, as stated in the *Muscle-Tendon Unit and Muscle Reflex Control* section, the reflex proportional gain is numerically related to the muscle activation, and muscle activation can be obtained by the processed sEMG signals. The left ankle reflex proportional gain used in the stiffness regulation loop can be obtained as follows (Pang et al., 2017):

(12) $$\begin{array}{l} K_{s}=K_{1}u_{1}+K_{2}u_{2} \end{array}$$

where *K*_1_ and *K*_2_ are the GAS stiffness and SOL stiffness, respectively; *u*_1_ and *u*_2_ are the corresponding muscle activation levels.

## Experimental Setup

**Figure 4:**
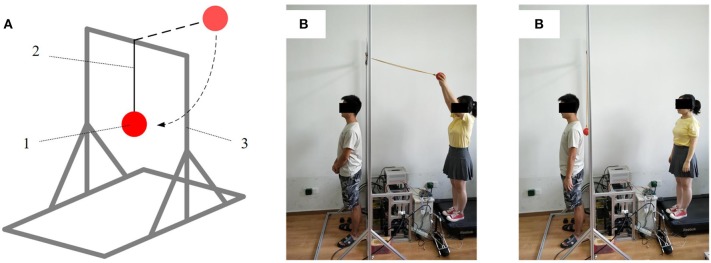
Experimental setup. **(A)** Structural schematic of experimental setup: (1) rubber ball, (2) connecting rope, (3) aluminum frame. **(B)** Photographic impression of experimental setup in operation.

**Figure 5:**
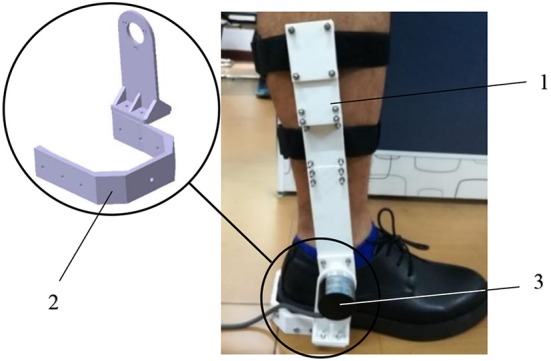
An assistant ankle angle measurement device: (1) shank bracket, (2) sole bracket, (3) optical incremental encoder.

The complexities and variations of the external environment lead to unexpected involvement of various reflexes, not least to muscle reflexes. In order to focus on muscle reflexes, it is paramount that experiments need to be conducted in a controlled procedure. To this end, as shown in Figure 4, this study designs a “Ball-disturbance” platform, in which a rubber ball (weight: 0.38 kg; diameter: 10 cm) is connected to an aluminum frame *via* a rope and released from the height of 2.5 m above the ground. Releasing from this given height, the ball impacts on the back of each subject to yield an external disturbance force. During the crash process, each subject is required to stand quietly. Moreover, the connecting rope is released from a tight initial state. Note that, based on our pilot test, by adopting the weight parameter and releasing height of the ball mentioned above, each subject could be guaranteed to lean within a tiny range (~-0.1–0.1 rad) and only muscle reflex control is included (Pang et al., 2017). In terms of the ankle angle data collection conducted in the pilot test, as visualized in Figure 5, an optical incremental encoder (NEMICON OVW2-36-2MD, resolution: 0.0008 rad) is fixed on the right ankle joint of each subject by two brackets and implemented as an assistant ankle angle measurement device to measure the subject's ankle angle. The e pressure sensor insoles (MOTICON) are placed in the subject's shoes for collecting ankle torque.

Based on the size and stiffness characteristics of the SERAJ, participants who were taller than 1.70 m, weighed <70 kg, and had an EU shoe size between 40 and 42 were selected. Therefore, six male subjects who have similar statures (mass 63.5 ± 2 kg, height 1.74 ± 0.03 m, thigh girth 0.42 ± 0.04 m, EU shoe size 42 ± 0.5) have been recruited in the experiment study. Each subject has signed a written informed consent proved by Wuhan University of Technology on the usage of humans as experimental participants for the experimental study prior to participating. Prior to conducting the human push-recovery experimental tests, the feasibility of proposed control algorithm on the designed SERAJ needs to be experimentally tested.

During the device experimental test, the SERAJ is horizontally fixed at one test-bed to ensure that the foot of this device is the only movable part. The foot is manually rotated within a certain range, creating ankle torque under the reflex control. The ankle angle and torque data of the device are collected by an optimal incremental encoder. After collecting these data of the device, they are sent to a laptop *via* a serial port to examine whether or not the ankle angle data and torque are linearly related to each other.

Aiming to test the response ability of the device to external disturbance, another experimental test needs to be conducted on the device with the control algorithm prior to the human-including push-recovery experimental test. In this experimental test, the SERAJ is also fixed the same way as described in the first device experimental test. Then, a small transient force is applied on the toe of the device. Again, the ankle angle and torque data of the device are collected through three optimal incremental encoders. Then, both of these two collected data are sent to a laptop to examine (1) whether or not the ankle angle and torque can be fast responsible to external disturbance; (2) whether or not the static errors of these two data can be acceptable.

During conducting the human push-recovery experimental tests, the SERAJ device is kneed on the right ankle of each subject and the “Ball-disturbance” platform described in Figure 4 is used to generate external disturbance on the back of each subject. A pressure sensor (DYMH-103) fixed to the ball's surface is utilized to collect the disturbance force. Three optical incremental encoders are, respectively, mounted on the motor output shaft, reducer output shaft, and robot ankle joint in SERAJ to collect the ankle motion angle and spring compression. The EMG (ELONXI EMG 100-Ch-Y-RA) with eight channels is used to collect the sEMG signals of SOL and GAS. Then, all the collected data are processed by STM32 controller with suitable sampling frequency (1 kHz). Moreover, a MATLAB custom software run on a laptop is programmed to record and analyze the data.

*T*-tests were used to verify differences in mean ankle angle, ankle torque, and muscle activation level between different conditions. Differences among subjects are not considered in this paper as joint-level dynamic variation commonly existed during movement. *p* < 0.05 is considered statistically significant for all tests.

## Experimental Results

In this section, experimental results are discussed from four perspectives to test the feasibility of the proposed reflex control algorithm. The four perspectives are (1) the relationship between the ankle torque and sway angle; (2) response faced with external disturbances; (3) ankle joint dynamic properties during upright standing push-recovery; and (4) influence on the contralateral ankle muscles. Note that the first two perspectives are carried out in the case where only the proposed control algorithm is inserted into the SERAJ without the subject being included. The last two perspectives are executed under the circumstance where the proposed control algorithm is inserted into the SERAJ with the subject being included.

### Relationship Between Ankle Torque and Sway Angle

**Figure 6:**
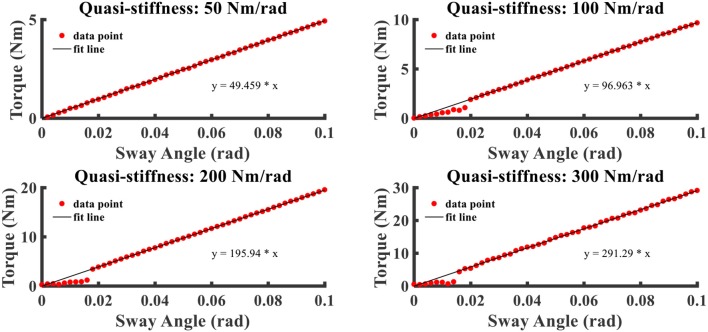
The relation curves that ankle torque varies with sway angle with different ankle joint quasi-stiffness settings.

Following the first device experimental test depicted in the *Experimental Setup* section, the relationship between ankle torque and sway angle under different stiffness settings is visualized in Figure 6. Four different quasi-stiffness values (50, 100, 200, and 300 Nm/rad) are tested to verify the design and control effect. One can make an observation from Figure 6 that the ankle torque is linearly related to the sway angle within a few tiny angles and the intensity of ankle torque depends on joint stiffness at the same angle, which, to some extent, can support the early assumption mentioned early in this paper. Moreover, the spring-like action characteristic can be proven through this experiment. Another point about that is the actual measured ratios of ankle movement to swag angle generally remain in line with the settings (*R*^2^ > 0.9) and the error is limited within 5%, which is a completely acceptable error. In other words, operation characteristics of the SERAJ can meet the anticipated effectiveness requirement in the “ball disturbance” push-recovery application.

### Responding to External Disturbance

**Figure 7:**
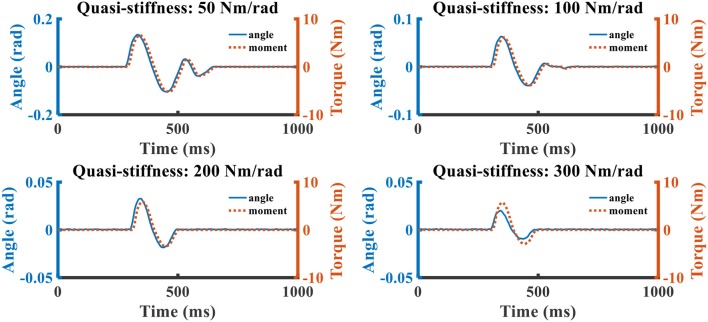
The relation curves that ankle torque and swag angle change over time. The blue solid line represents the sway angle, and the red dotted line is the joint torque.

The dynamic responses of SERAJ to external disturbance controlled with different reflex proportion parameters are depicted in Figure 7. The unit of the proportion parameter is defined as Nm/rad because we assume a linear relationship between muscle activation and muscle force in upright stance (Pang et al., 2019) and the gain of muscle activation to muscle force is multiplied implicitly in the reflex parameters. The prefix “quasi” in the figures means to distinct from the mechanical stiffness as the “reflex stiffness” behavior is controlled by algorithm (Rouse et al., 2013).

With the increasing of quasi-stiffness, the response oscillation and peak–peak joint deviation amplitude are attenuated. When the quasi-stiffness is set as 300 Nm/rad, the foot of the SERAJ device first vibrates twice about original position and the magnitude of ankle joint angle is about 0.025 rad when the external force is acted on the device. Then, the disturbed device can return to the original balance state around in 200 ms without deviation. This implies that the SERAJ can fast reply to the external disturbance with a zero static error when the device is controlled by our proposed control algorithm. This, to a certain degree, can further confirm the feasibility of the proposed method on the device in the case where no subject is involved.

### Ankle Joint Dynamic Properties During Upright Standing Push-Recovery

**Figure 8:**
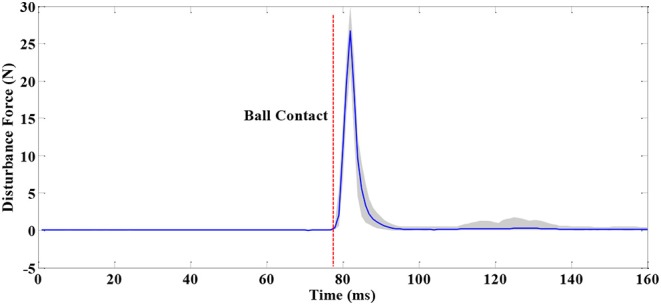
Disturbance force in “Ball-disturbance” trials (where the blue line and gray-shaded area represent the average and standard deviation of measurement results of many experiments, respectively).

**Figure 9:**
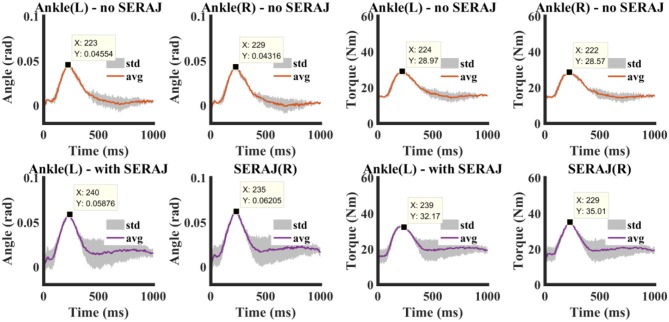
Experimental data of a representative subject in “Ball-disturbance” trials. Upper and lower panels show experimental results without and with SERAJ, respectively (where the red/purple lines and the gray-shaded areas represent the average and standard deviation of measurement results of experiments, respectively). Maximum values of each curve are attached in the panel.

The “ball-disturbance” exerts an impulse-like perturbation (as shown in Figure 8) with amplitude of about 25 N and duration of about 10 ms to the subject. The experimental results of ankle joint sway angles and torques of subject C are depicted in Figure 9. The start point of the time interval is set as 50 ms before disturbance arrives, and the length of the time interval is selected as 1 s as it is long enough for subjects to complete the task. The features to describe “ball-disturbance” push-recovery are defined as duration of leaning forward and return back, peak angle of left and right ankle, and peak torque of left and right ankle in this paper.

It can be seen that the body of the subject starts to move back to the balance position after the sway angle reaches about 0.045 rad when SERAJ is not mounted. The maximum lean forward angle of the left ankle increases to 0.058 when SERAJ is mounted on the right knee. The same peak amplitude increase induced by SERAJ in ankle torque (28.5 Nm vs. 35.1 Nm) can be found. The increasing trend is significant (*p* < 0.05) in all the five subjects except subject A. There are significant differences in leaning forward duration (*p* = 0.026) and return back duration (*p* = 0.037) between SERAJ unmounted and mounted cases for subject C. However, the correlation values of leaning forward curve and return back curve between SERAJ unmounted and mounted case are 0.91 and 0.87, respectively. These high correlation values indicate that the trends of leaning forward when SERAJ is mounted are similar to the trend when SERAJ is not mounted. The correlation values of leaning forward and return back are relatively high among all the six subjects (as shown in Table 4). Experimental results of all six subjects are listed in Table 3 in the form of mean ± SD.

**Table 3:** The experimental results of all the six subjects.

**Subject**					**Peak angle (rad)**				**Peak torque (Nm)**			
	**Lean forward time (ms)**		**Turn back time (ms)**		**Left ankle**		**Right ankle/SERAJ**		**Left Ankle**		**Right ankle/SERAJ**	
	**No SERAJ**	**With SERAJ**	**No SERAJ**	**With SERAJ**	**No SERAJ**	**With SERAJ**	**No SERAJ**	**With SERAJ**	**No SERAJ**	**With SERAJ**	**No SERAJ**	**With SERAJ**
A	226 ± 1	244 ± 7	320 ± 54	259 ± 61	0.069 ± 0.006	0.086 ± 0.004	0.066 ± 0.005	0.090 ± 0.006	36.43 ± 2.18	42.00 ± 1.67	36.60 ± 2.89	45.01 ± 1.48
B	222 ± 7	232 ± 1	285 ± 74	227 ± 45	0.064 ± 0.010	0.069 ± 0.011	0.064 ± 0.010	0.075 ± 0.011	35.86 ± 3.82	36.70 ± 3.59	35.43 ± 3.03	39.53 ± 4.58
C	226 ± 1	238 ± 11	331 ± 4	253 ± 21	0.046 ± 0.001	0.059 ± 0.001	0.043 ± 0.002	0.062 ± 0.002	28.97 ± 0.57	32.17 ± 0.54	28.57 ± 0.65	35.01 ± 0.04
D	223 ± 8	228 ± 20	411 ± 14	384 ± 11	0.027 ± 0.001	0.048 ± 0.006	0.025 ± 0.001	0.052 ± 0.004	23.02 ± 0.16	29.54 ± 1.69	22.78 ± 0.19	31.44 ± 1.75
E	210 ± 10	237 ± 4	248 ± 36	183 ± 21	0.063 ± 0.015	0.077 ± 0.016	0.060 ± 0.014	0.080 ± 0.018	35.20 ± 4.33	39.34 ± 5.78	34.69 ± 4.36	41.46 ± 6.12
F	239 ± 9	221 ± 3	226 ± 21	215 ± 30	0.052 ± 0.011	0.061 ± 0.006	0.052 ± 0.009	0.065 ± 0.004	31.11 ± 2.60	33.71 ± 0.78	30.59 ± 2.57	35.97 ± 1.97

**Table 4:** Muscle activations of left ankle during the experiment (GAS, gastrocnemius muscle; SERAJ, series elastic robot ankle joint; SOL, soleus muscle).

**Subject**	**Left GAS**		**Left SOL**	
	**No SERAJ**	**With SERAJ**	**No SERAJ**	**With SERAJ**
A	0.55 ± 0.15	0.57 ± 0.11	0.57 ± 0.01	0.58 ± 0.09
B	0.41 ± 0.35	0.31 ± 0.21	0.48 ± 0.13	0.53 ± 0.12
C	0.44 ± 0.05	0.59 ± 0.02	0.52 ± 0.05	0.59 ± 0.04
D	0.28 ± 0.02	0.35 ± 0.21	0.44 ± 0.06	0.48 ± 0.18
E	0.50 ± 0.22	0.54 ± 0.15	0.55 ± 0.03	0.54 ± 0.05
F	0.50 ± 0.11	0.64 ± 0.04	0.59 ± 0.04	0.55 ± 0.01

### Muscle Activation on Contralateral Ankle Muscles

**Figure 10:**
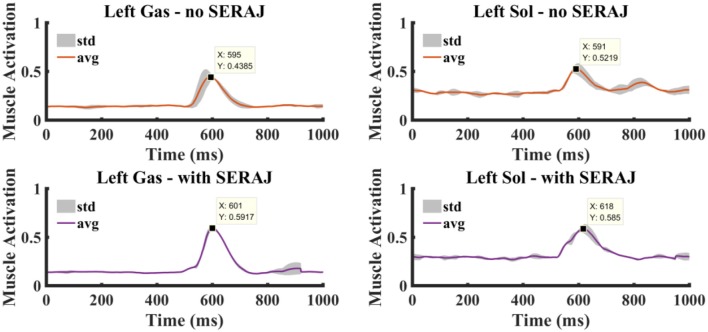
The muscle activation curves at the left ankle joint. Right column shows the gastrocnemius muscle activation without and with the series elastic robot ankle joint (SERAJ) kneed on the subject's right ankle. Left column shows the soleus muscle activation without and with the SERAJ kneed on the subject's right ankle. Solid lines represent average values, and gray-shaded areas show standard deviation.

The purpose of the exoskeleton device is to enhance the human joint, whereas the prosthesis intends to replace the joint and replicate the original biomechanical function. So, the question remains: Is there an additional effect on the subject's contralateral ankle motion with the use of SERAJ? Interestingly, sEMG as the electrical manifestation of muscle contraction contains rich information for decoding motion intention, including the simultaneous recognition of both motion types and developed force (Au et al., 2007; Darak and Hambarde, 2015). So different sEMG signals are collected in two groups of experiments performed in the platform introduced in the *Experimental Setup* section to address the concern. In the first group, a subject (subject C) alone, without the SERAJ kneed on his right ankle, is impacted by a rubber ball from 2.5 m height on the back. The sEMG signals of the subject's left ankle muscle are measured to analyze muscle activation. In the second group, the same subject repeats the experiment mentioned above in equal conditions except that the SERAJ is kneed on the right ankle this time. The activation of the subject's left ankle muscle also is analyzed to compare it with the last experimental result, as shown in Figure 10.

Compared with no SERAJ case, muscle activations of Gas and Sol averagely increase significantly (Gas: *p* << 0.05, Sol: *p* << 0.05) by 0.13 and 0.06, respectively. As the same in sway angle and ankle torque situation, correlation values of Gas and Sol between SERAJ mounted and unmounted are close to 1 (0.91 and 0.92, respectively). The increased trend can be found in all the six subjects, except that Gas activation value decreased in SERAJ mounted case for subject B. It can be indicated that subjects intend to activate contralateral joint muscles (the left ankle) more when the right ankle is replaced by SERAJ, whereas the activation dynamic profiles trend keep the same. All the six subjects muscle activation values are listed in Table 4 in the form of mean ± SD.

## Discussion

Ankle joint active reaction to perturbation is important to maintain an upright stance postural. The movement of amputees with passive ankle joint is heavily limited, and the intact limbs have to provide more compensation and cost more metabolic energy to realize standing balance. The experimental results show that our proposed muscle reflex algorithm can achieve upright standing push-recovery movement on a unilateral mounted SEA-based ankle joint emulator. The “Ball release” task aims to mimic the common but not easy to perceptible disturbance in daily lives, which can break the balance if there is only intrinsic stiffness.

The torque control ability within tiny angles indicates that the cable-driven design is valid and SERAJ can produce accurate torque commanded by the reflex controller within a human upright standing sway range. It is vital for powered ankle prosthesis to regulate torque precisely in a relatively small range (2–5°) because the base of support of human is limited in foot-in-place upright stance (Hof and Curtze, 2016). The quasi-stiffness of 300 Nm/rad for one ankle joint is similar to the “reference stiffness” (Vlutters et al., 2015) of a person with 60 kg weight, which verifies the ability of SERAJ to provide enough resistance torque to keep balance. The capability to regulate variable stiffness is necessary for a powered ankle prosthesis as human adjusts joint intrinsic stiffness or quasi-stiffness to achieve different tasks when interacting with the physical environment. Although the movement range is not large for ankle joint in upright stance, the variable quasi-stiffness is also needed as the intrinsic stiffness of each individual is different in standing postural.

The results conducted from the external disturbance experimental tests indicate that the reaction frequency of SERAJ is around 3 Hz, which is analogous to the human joint (Hogan, 2017). For a unilateral mounted prosthesis, it is helpful to regulate the reaction response to be consistent with the contralateral intact lower limb to fulfill a cooperation movement. Too fast or too slow motion of robotic device would make a conflict with the intact limb and bring another disturbance to the torso. The conventional trajectory following method, such as position (Scherillo et al., 2003) or impedance trajectory (Dhir et al., 2018), is not suitable in standing push-recovery task because there is no obvious rhythm motion and torque–angle relationship varies with unpredictable external disturbance. The impedance control-based algorithm is preferred in such a case. An impedance controller is designed for a powered ankle–foot orthosis to maintain upright standing balance (Emmens et al., 2018). The variables in the controller are the COM position and velocity, which are acquired by a four-link human body model and the impedance parameters are constant. For an exoskeleton device or orthosis, it is not required to replace the original body part to fulfill the task, whereas the prosthesis has to reproduce the full motion function or provide enough support, passively or actively, to realize a stable motion. The human-like response of SERAJ is achieved by the combination of the muscle reflex controller, whose form and parameters are designed referred to the ones of human, and the compressed spring, which forms the intrinsic stiffness. The stretch or PD parameters are regulated online by calculated muscle activation levels of contralateral ankle joint muscles. As constant impedance is not enough to keep upright balance for human, the controller has to be able to compensate the required additional torque to stop body leaning forward trend. Taking the contralateral joint dynamic property as the reference (we adopt a linear relationship between muscle activation and joint stiffness) is helpful, as shown by the experimental results, to realize a stable prosthesis-to-intact limb cooperation work. The linear relationship is similar with the reported behavior of human that ankle joint intrinsic stiffness increases linearly with ankle joint sway angle (Amiri and Kearney, 2019), which is helpful to guarantee postural stability as the center of pressure moves toward the edge of the base of support. The intrinsic stiffness which is similar to the one of human joint and much lower than traditional robotic system, is also necessary in prosthesis design, like SERAJ. Besides the advantages of compliance in joint, such as energy efficiency and impact protection, it is the fundamental part forming human dynamic behavior. Although the compliance property can be realized by control algorithm (Calanca et al., 2016), the controlled behavior may be unable to match with the natural response of the physical system as the response frequency of the actuator is limited.

The experimental results show that activations of SOL and GAS in two cases all feature the similar profile, but activation levels of SOL and GAS have upward trends when wearing SERAJ although subject's ankle joint angle and torque are similar (almost the same range of ankle angle and ankle torque) in two conditions. The same profiles of left ankle joint indicate that the dynamic behavior of the SERAJ performed during push-recovery task is similar with the right ankle. As a consequence, the proposed muscle reflex control realized on SERAJ could be analogous to the human behavior intention. The increasing muscle activation and joint torque imply that contralateral ankle output more work when wearing SERAJ. Subjects seem to intend to rely on their own ankle which can be controlled by themselves. Due to several objective factors, such as SERAJ's weight, which is not quite identical to the own leg of subject, and how the SERAJ is worn, there is no doubt that subject's left ankle could have to face extra burden that can be seen from the difference of activation level in the same muscle for upright push-recovery when wearing SERAJ. Most likely due to the higher activation level of the left ankle, the torque of the left ankle joint built up more rapidly and greater when wearing SERAJ. Moreover, subjects tend to utilize muscle synergy to coordinate muscles to keep balance, in which sensory information from whole body are input for the CNS. In our experimental protocol, each subject is required to knee on SERAJ. This may influence the sensation system as the right foot of each subject is suspended. As a consequence, each subject may adopt an alternation synergy in which the left ankle muscles are activated more.

The drawback of SERAJ structure is that electric motor is hanging on the leg part which removes the inertia of SERAJ from the vertical line of the subject's thigh. However, the effect is limited to our experiments as the push is in a sagittal plane and the entire weight of SERAJ is smaller than that of each subject's leg. Moreover, the target of this study is to test the proposed muscle reflex controller, rather than a novel prosthesis design used in daily life. The improvement of the device can be considered as an extension of this study in the near future.

## Conclusion

In this study, a specialized muscle reflex control for robot ankle joint is presented to complete upright standing push-recovery during “ball-disturbance.” The dynamic properties of the unmounted SERAJ ankle joint are analyzed to assess the feasibility of the control. Experiments have shown that the dynamic performance, especially the trend of the dynamic profile of the contralateral ankle joint is almost consistent before and after using SERAJ. As many studies focus on large disturbance push-recovery using exoskeleton device or under locomotion circumstances, our results show that the proposed reflex control algorithm can guarantee upright standing balance using unilateral powered ankle joint prosthetics in ankle strategy situation, which is a common situation during daily living.

## Data Availability Statement

The raw data supporting the conclusions of this article will be made available by the authors, without undue reservation, to any qualified researcher.

## Author Contributions

YC made a contribution to building up a framework of muscle reflex control. KX made a contribution to designing a series elastic actuator. BT made a contribution to guiding experiments. ZJ made a contribution to analysis of experimental data. MP made a contribution to building upright standing push-recovery model with reflex control.

## Conflict of Interest

The authors declare that the research was conducted in the absence of any commercial or financial relationships that could be construed as a potential conflict of interest.

## Footnotes

^1^https://simtk-confluence.stanford.edu/display/OpenSim/Musculoskeletal+Models#MusculoskeletalModels-OpenSimCoreModels
